# PD-L1 expression combined with microsatellite instability/CD8+ tumor infiltrating lymphocytes as a useful prognostic biomarker in gastric cancer

**DOI:** 10.1038/s41598-019-41177-2

**Published:** 2019-03-15

**Authors:** Toshiaki Morihiro, Shinji Kuroda, Nobuhiko Kanaya, Yoshihiko Kakiuchi, Tetsushi Kubota, Katsuyuki Aoyama, Takehiro Tanaka, Satoru Kikuchi, Takeshi Nagasaka, Masahiko Nishizaki, Shunsuke Kagawa, Hiroshi Tazawa, Toshiyoshi Fujiwara

**Affiliations:** 1 Department of Gastroenterological Surgery, Okayama University Graduate School of Medicine, Dentistry and Pharmaceutical Sciences, Okayama, Japan; 2 Center for Innovative Clinical Medicine, Okayama University Hospital, Okayama, Japan; 3 Department of Pathology, Okayama University, Okayama, Japan; 4 Minimally Invasive Therapy Center, Okayama University Hospital, Okayama, Japan; 5 Department of Clinical Oncology, Kawasaki Medical School, Kurashiki, Japan

## Abstract

While the importance of programmed death-ligand 1 (PD-L1), mutation burden caused by microsatellite instability (MSI), and CD8+ tumor infiltrating lymphocytes (TILs) has become evident, the significance of PD-L1 expression on prognosis still remains controversial. We evaluated the usefulness of combined markers of PD-L1 and MSI or CD8+ TILs as a prognostic biomarker in gastric cancer. A total of 283 patients with gastric cancer were reviewed retrospectively. PD-L1 expression on >5% tumor cells was defined as PD-L1-positive. PD-L1-positive rate was 15.5% (44/283). PD-L1 positivity was significantly correlated with invasive and advanced cancer and also significantly correlated with MSI, whereas no significance was observed with CD8+ TILs. Kaplan–Meier analysis showed that PD-L1 positivity significantly correlated with a poor prognosis (p = 0.0025). Multivariate analysis revealed that PD-L1 positivity was an independent poor prognostic factor (hazard ratio [HR]: 1.97, p = 0.0106) along with diffuse histological type and lymph node metastases. Combinations of PD-L1 and MSI (HR: 2.18) or CD8+ TILs (HR: 2.57) were stronger predictive factors for prognosis than PD-L1 alone. In conclusion, combined markers of PD-L1 and MSI or CD8+ TILs may be more useful prognostic biomarkers in gastric cancer, and better clarify the immune status of gastric cancer patients.

## Introduction

Immunotherapy has become popular in the field of cancer therapy worldwide, especially since cancer immunotherapy was named “Breakthrough of the Year” by Science magazine in 2013^1^, mainly due to success in immune-checkpoint blockade therapy. One of the most common mechanisms underlying immunotherapy is programmed death-1 (PD-1) and programmed death-ligand 1 (PD-L1). The PD-1/PD-L1 interaction, which works as an inhibitory factor in the last step of the cancer immunity cycle, induces functional impairment of antigen-specific T cells, leading to immune evasion by tumors^2,3^. PD-1/PD-L1 blockade therapy with antagonistic antibodies has achieved great success in clinical trials for patients with various types of cancer, and has been approved for use in clinical practice for patients with several types of cancer including gastric cancer^4–10^.

The importance of PD-L1 expression on solid tumors as a prognostic biomarker has been reported in multiple studies, many of which have noted the association between PD-L1 expression in tumor tissues and a worse prognosis, although this association varied according to tumor type^11^. In gastric cancer, which is the third leading cause of cancer-related death and fifth most common malignancy worldwide^12^, PD-L1 overexpression in tumor tissues tends to be associated with a worse prognosis^13–15^, although some reports have noted that overexpression correlates with a better prognosis^16,17^. Thus, the association between PD-L1 expression and prognosis in gastric cancer still remains a subject of debate.

The Cancer Genome Atlas (TCGA) Research Network recently proposed a novel classification in which gastric cancer is divided into four subtypes based on the underlying molecular biology; this is expected to provide a new roadmap for patient stratification and treatment strategy in gastric cancer^18^. In this classification, status of microsatellite instability (MSI) and Epstein-Barr virus (EBV), both of which are well recognized as factors associated with the tumor immune environment, was each focused as one of key factors to divide into four subtypes. MSI refers to the hypermutatable state of cells caused by impaired DNA mismatch repair, and MSI-high cancer has an increased number of mutation-associated neoantigens, leading to stimulation of anti-tumor immunity^19^. EBV-positive gastric cancer, which reportedly accounts for about 8.8% of all gastric carcinoma, has also received attention as a tumor population with elevated PD-L1 expression^20^.

In this study, we evaluated PD-L1 expression and status of MSI, EBV, and tumor-infiltrating lymphocytes (TILs) on gastric cancer tissues which were surgically removed, and assessed the correlation of PD-L1 expression with the status of these factors and the prognosis of patients. We further investigated the usefulness of PD-L1 and MSI or TIL as a combined marker to better predict prognosis than a single marker of PD-L1. While the importance of each factor as a prognostic or predictive biomarker for anti-PD-1 therapy has been extensively reported, to the best of our knowledge, there have been few reports on these combined markers. The results of this study may help with the evaluation of tumor status from the aspect of tumor immunity in gastric cancer, which could lead to the development of more reasonable and effective treatment strategies including immune checkpoint inhibitors for gastric cancer.

## Results

**Figure 1:**
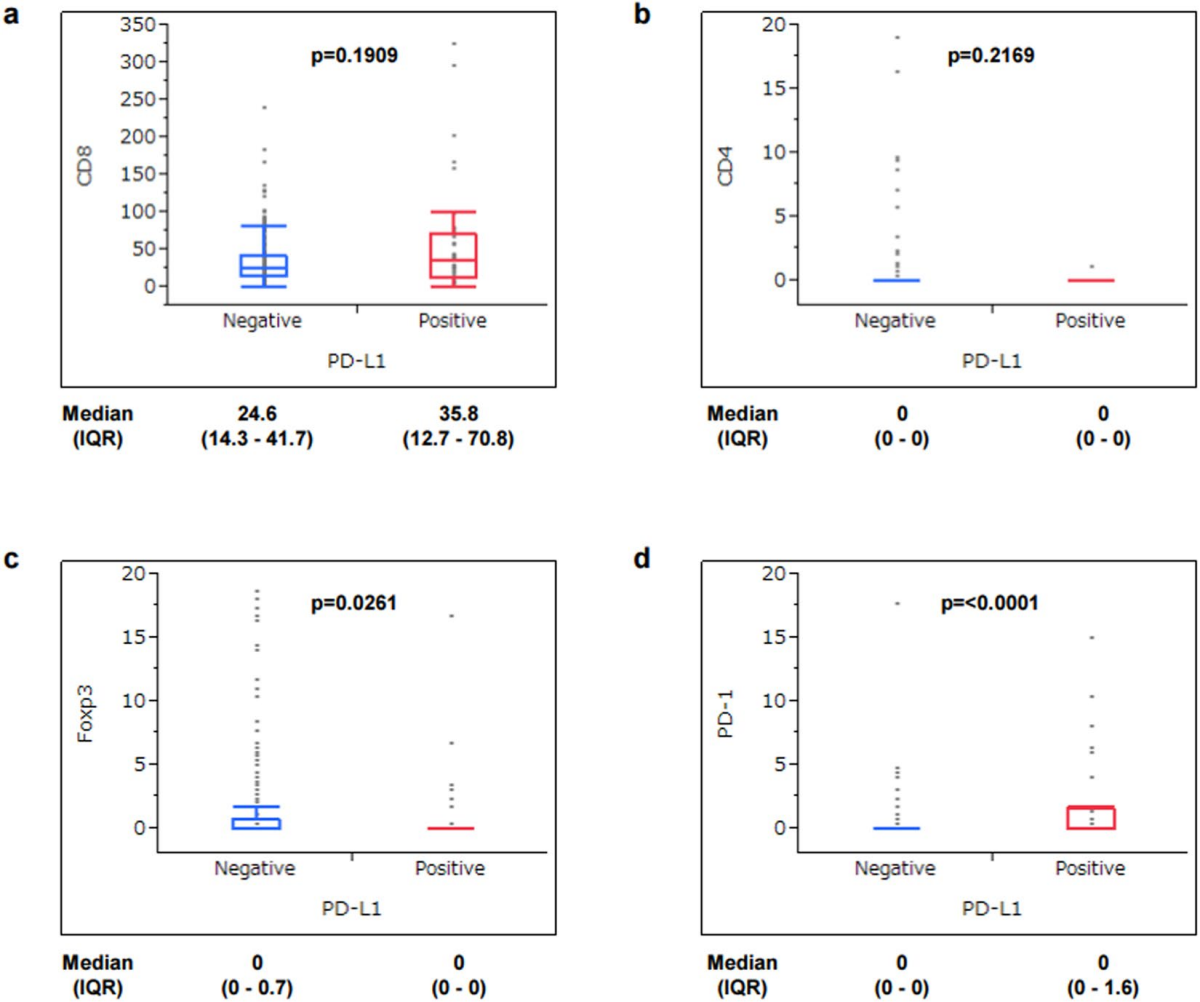
Correlation of PD-L1 expression with TIL surface markers. Median value of TILs with expression of CD8 (**a**), CD4 (**b**), Foxp3 (**c**), and PD-1 (**d**) was calculated from counting on three different fields of immunohistochemical staining, and classified into two groups (positive and negative) based on each cutoff value. The cutoff value was set at 20 for CD8, 1 for CD4, 1 for Foxp3, and 1 for PD-1. IQR, interquartile range

The correlation of PD-L1 expression with clinicopathological characteristics, including status of MSI, EBV, and TILs expressing CD8, CD4, Foxp3, and PD-1, was determined. Based on our evaluation criteria of PD-L1 expression, the PD-L1-positive (score 2+ and 3+) rate was 15.5% (44/283) while the PD-L1-negative rate (score 0 and 1+) was 84.5% (239/283). MSI was observed in 7.8% (22/283) and EBV was detected in 8.1% (23/283). Table 1 shows the correlation of PD-L1 expression with clinicopathological findings. PD-L1 positivity was observed in more male patients (p = 0.0187). In histological findings, PD-L1 positivity was observed more in intestinal type than in diffuse type (p = 0.0201), and in invasive and advanced cases based on status of ly (p = 0.0014), v (p = 0.0154), T (p = 0.0063), and stage (p = 0.0258). PD-L1 positivity tended to be observed more in tumors located in the upper third of the stomach (p = 0.0660). PD-L1 positivity was significantly correlated with MSI (p = 0.0006) whereas no correlation was observed with EBV (p = 0.3926). With respect to TILs, while CD8 and CD4 expression was not correlated with PD-L1 expression (Fig. 1a,b), Foxp3 positivity was more observed in PD-L1-negative tumors (p = 0.0261) (Fig. 1c) and PD-1 positivity was more observed in PD-L1-positive tumors (p < 0.0001) (Fig. 1d).

**Table 1:** Clinicopathological characteristics of patients with PD-L1-negative and PD-L1-positive gastric cancer.

	PD-L1		p value
	Negative (n = 239)	Positive (n = 44)	
Age (years)	66.5 ± 11.8	68.9 ± 8.8	0.2009
Sex			
male	152 (64%)	36 (82%)	0.0187
female	87 (36%)	8 (18%)	
Histological type			
intestinal	123 (51%)	31 (70%)	0.0201
diffuse	116 (49%)	13 (30%)	
Lymphatic invasion (ly)			
negative	78 (33%)	4 (9%)	0.0014
positive	159 (67%)	40 (91%)	
Venous invasion (v)			
negative	117 (49%)	13 (30%)	0.0154
positive	120 (51%)	31 (70%)	
Tumor location			
upper third	51 (21%)	15 (34%)	0.0660
middle or lower third	188 (79%)	29 (66%)	
Depth of tumor invasion (T)			
1	95 (40%)	8 (18%)	0.0063
2, 3, 4	144 (60%)	36 (82%)	
Lymph node metastasis (N)			
0	126 (53%)	17 (40%)	0.0946
1, 2, 3	110 (47%)	26 (60%)	
Stage			
I	110 (46%)	12 (28%)	0.0258
II, III, IV	128 (54%)	31 (72%)	
MSI			
non-MSI	226 (95%)	35 (80%)	0.0006
MSI	13 (5%)	9 (20%)	
EBV			
negative	221 (92%)	39 (89%)	0.3926
positive	18 (8%)	5 (8%)

### Impact of PD-L1 expression on overall survival

**Figure 2:**
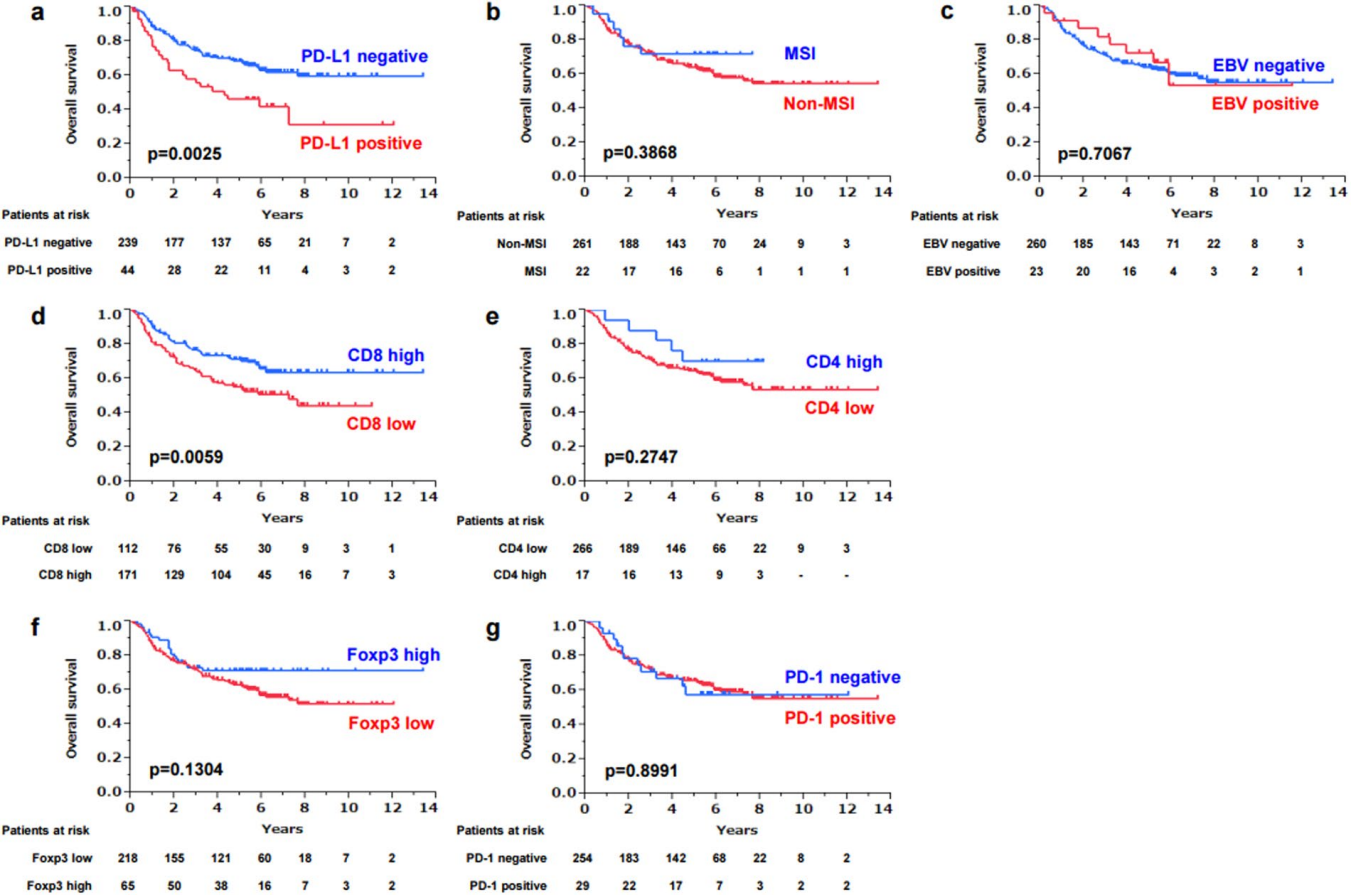
Kaplan–Meier survival curve of gastric cancer patients based on PD-L1 expression on tumor cells (**a**), MSI status (**b**), EBV positivity (**c**) and expression on TILs of CD8 (**d**), CD4 (**e**), Foxp3 (**f**) and PD-1 (**g**). Censored cases are shown as tick marks in each graph.

In this study, 104 of 283 patients died, including 65 patients who died of gastric cancer and 39 patients who died of other diseases. PD-L1 positivity was correlated with a significantly poor prognosis compared to PD-L1 negativity (p = 0.0025) (Fig. 2a), whereas MSI and EBV did not have much influence on overall survival (OS) (Fig. 2b,c). On analysis of 106 patients who had lymph node metastasis, the PD-L1-positive rate was 15% (16/106) on primary tumors and 13% (14/106) on metastatic lymph nodes (Supplementary Table S1). On these 106 patients, PD-L1 expression on metastatic lymph nodes did not make much difference on OS, whereas PD-L1-positive expression on primary tumors caused a significantly poor prognosis (Supplementary Fig. S1), which suggested that PD-L1 expression on primary tumors likely influenced OS more strongly than PD-L1 expression on metastatic sites. When OS was analyzed based on the combination of PD-L1 expression on primary tumors and metastatic lymph nodes, PD-L1 negativity seemed to correlate with a better prognosis (p = 0.0459) than the other populations (Supplementary Fig. S2). Among the four surface markers of TILs examined in this study, only CD8 had a significant effect on OS. CD8 low correlated with a poorer prognosis (p = 0.0059) (Fig. 2d), whereas CD4, Foxp3, and PD-1 did not have a significant difference on OS (Fig. 2e–g). Univariate and multivariate analyses of factors related to OS showed that PD-L1 positivity was an independent poor prognostic factor (hazard ratio [HR]: 1.97, 95% confidence interval [CI]: 1.18–3.20, p = 0.0106) along with diffuse histological type and the presence of lymph node metastasis (Table 2).

**Table 2:** Univariate and multivariate analyses of factors related to overall survival.

	Univariate	Multivariate		
	p value	Hazard ratio	95% CI	p value
Age (years) (≥70/<70)	0.0741			
Sex (female/male)	0.9490			
PD-L1 (positive/negative)	0.0053	1.97	1.18–3.20	0.0106
MSI (non-MSI/MSI)	0.3636			
EBV (positive/negative)	0.7015			
CD8 (high/low)	0.0068			0.0737
CD4 (high/low)	0.2434			
Foxp3 (low/high)	0.1173			
PD-1 (positive/negative)	0.8996			
Histological type (diffuse/intestinal)	0.0008	2.23	1.46–3.43	0.0002
Lymphatic invasion (positive/negative)	<0.0001			0.4400
Venous invasion (positive/negative)	<0.0001			0.0516
Tumor location (U/M, L)	0.0497			0.0968
Depth of tumor invasion (T) (2, 3, 4/1)	<0.0001			0.1014
Lymph node metastasis (N) (1, 2, 3/0)	<0.0001	2.09	1.23–3.74	0.0058

### Combined marker of PD-L1 expression with MSI or CD8+ TILs

**Figure 3:**
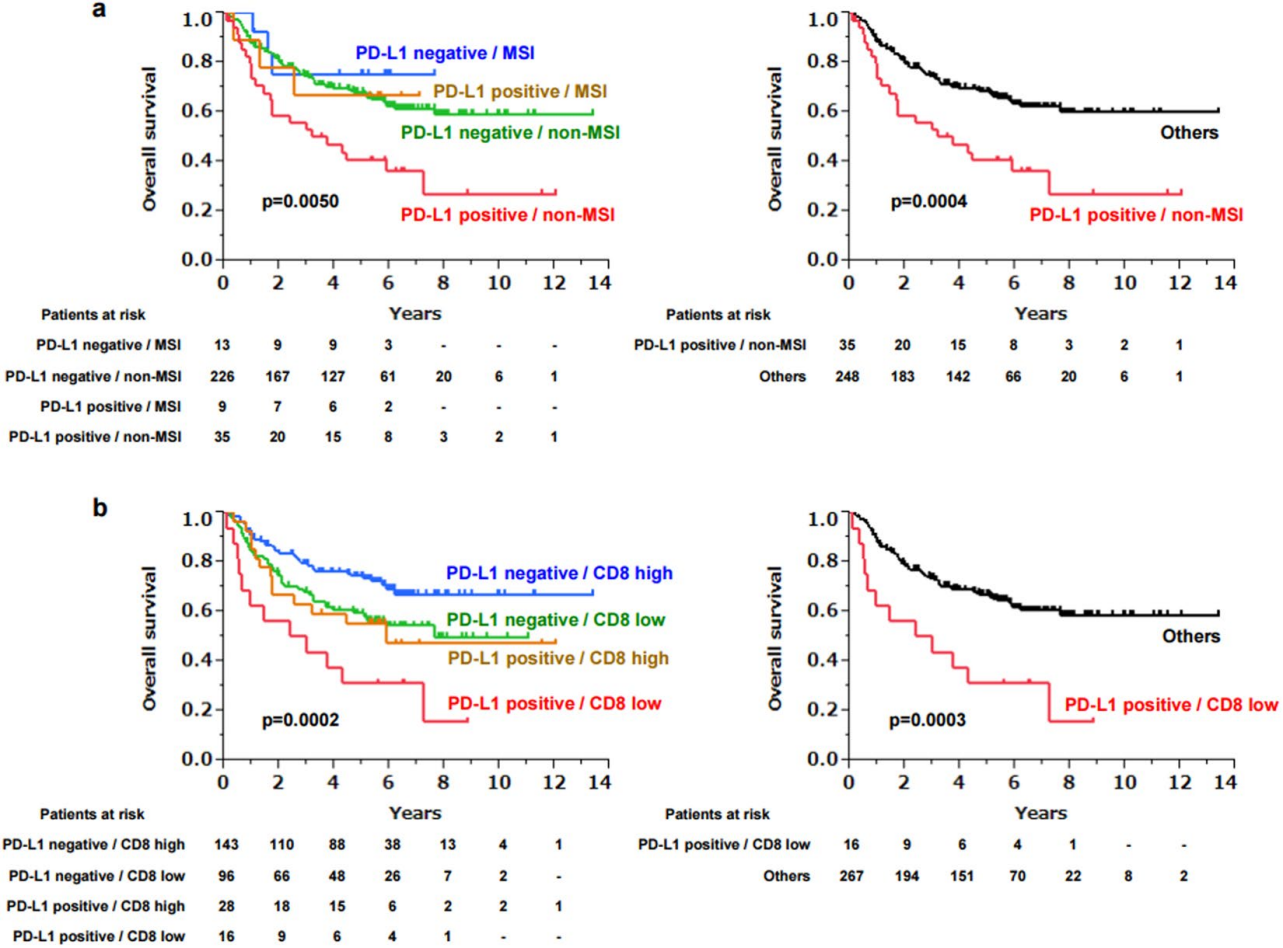
Kaplan–Meier survival curve of gastric cancer patients based on the combined markers of PD-L1 and MSI (**a**), and PD-L1 and CD8 (**b**). In the right graph, three groups other than “PD-L1 positive/non-MSI” in (**a**) or “PD-L1 positive/CD8 low” in (**b**) were combined as “others”. Censored cases are shown as tick marks in each graph.

When Kaplan–Meier survival analysis was performed on four groups divided based on each status of PD-L1 expression and MSI, patients with PD-L1 positivity and non-MSI had a significantly poor prognosis (p = 0.0050), whereas the other three groups had a similar prognosis (Fig. 3a). The same results were observed with the combination of PD-L1 and CD8+ TILs, as patients with PD-L1 positivity and CD8 low had a significantly poor prognosis compared to the other populations (p = 0.0002) (Fig. 3b). To compare the impact of these combined markers to a single marker of PD-L1 on prognosis, the same univariate and multivariate analyses performed in Table 2 were performed, in which PD-L1 was replaced with either PD-L1/MSI or PD-L1/CD8. This multivariate analysis revealed that PD-L1-positive/non-MSI and PD-L1-positive/CD8 low were both independent poor prognostic factors, and interestingly, the HRs of PD-L1-positive/non-MSI (2.18) and PD-L1-positive/CD8 low (2.57) were higher than that of PD-L1-positive alone (1.97) (Table 3), which suggested that the combined markers of PD-L1 with MSI or CD8 were more important and useful as a prognostic biomarker than a single marker of PD-L1.

**Table 3:** Multivariate analysis of PD-L1 combined with MSI or CD8 status on overall survival.

	Multivariate		
	Hazard ratio	95% CI	p value
PD-L1	1.97	1.18–3.20	0.0106
PD-L1/non-MSI	2.18	1.28–3.58	0.0050
PD-L1/CD8 low	2.57	1.28–4.79	0.0095

Factors of histological type, lymphatic invasion, venous invasion, tumor location, depth of tumor invasion (T) and lymph node metastases (N) with each PD-L1-related factor described above were subject to multivariate analysis.

## Discussion

It has become widely recognized that the interaction of PD-1 and PD-L1 plays an important role in immune evasion by tumors, and PD-L1 expression on tumor tissues may be a good biomarker to predict the efficiency of anti-PD-1/PD-L1 antibodies^21^. The importance of PD-L1 expression on tumor tissues as a prognostic factor has also been reported in many studies^11^. However, the issue remains that the evaluation criteria of PD-L1 expression is not standardized, for example, it remains unknown which antibody to use. Staining pattern and intensity vary according to the difference of antibody and the method of staining^22^. We employed a PD-L1 antibody of E1L3N in this study because it was considered the most appropriate staining to use to evaluate PD-L1 expression based on the preliminary comparison of three different antibodies to human PD-L1 (E1L3N, 28–8 and SP142) prior to initiation of this study. It is also unknown which area of tumor tissues should be evaluated, and which type of cells (i.e., tumor cells, immune cells, stromal cells). In this study, we simply focused on PD-L1 expression on tumor cells, but the importance of PD-L1 expression on stromal cells or immune cells such as dendritic cells and macrophages was recently reported^23^. The appropriate cutoff value of PD-L1 expression is also unknown. Percentages of 1%, 5%, 10%, and even 50% were reportedly used as a cutoff value of PD-L1 expression^24^. Although the most appropriate cutoff value is not determined yet, a higher cutoff value seems to better predict the prognosis of patients and the efficacy of anti-PD-1/PD-L1 antibodies. Actually in a clinical trial, PD-L1 expression in at least 50% of tumor cells in non-small cell lung cancer tissue was correlated with better efficacy of pembrolizumab than one in less than 50%^25^. In this study, we classified PD-L1 expression into four groups, and PD-L1 positivity was an independent poor prognostic factor when 5% was selected as a cutoff value. However, when 1% was set as a cutoff value, no significant difference was observed between PD-L1 positive and negative.

The histologic and biologic backgrounds of gastric cancer differ between East and West^26^. In Asia, gastric cancer is characterized by a diffuse-type histology, tumors primarily located in the distal stomach, and a correlation with *Helicobacter pylori* status, in contrast to the disease in Western countries. The TCGA project of gastric cancer highlighted MSI and EBV as key molecules to divide gastric cancers into four subtypes^18^, which has significantly impacted actual clinical practice and basic research in Asia, even though approximately 75% of patients in the TCGA project were from Western countries. EBV was found in 8.8% of 295 gastric cancers and was associated with amplification of PD-L1 in the TCGA report. In our case, EBV was not correlated with PD-L1 expression, while EBV positivity (8.1%) was similar with the TCGA report. MSI seems to have a stronger connection with PD-L1 expression and PD-1/PD-L1 blockade therapy as evidenced by the fact that pembrolizumab was approved for any types of solid tumor with MSI^27^. While the MSI-high population in this study (7.8%) was a little smaller than that in the TCGA study (21.7%) and other previous studies (11.68–33.82%)^28^, MSI was significantly correlated with PD-L1 positivity, and more importantly, the combination of PD-L1 and MSI status was a stronger prognostic marker than a single marker of PD-L1. Another main characteristic in cancer immunity was CD8+ TILs, and the clinical significance of TILs has been reported in many studies of gastric cancer^29^. In our study, tumors with low CD8+ TILs showed a significantly worse prognosis, and combination of PD-L1 and CD8 was also found to be a useful prognostic marker.

With respect to the correlation of PD-L1 with other TIL markers, PD-1 expression on TILs was significantly correlated with PD-L1 expression on tumor cells in this study. This is expected because PD-L1 is a ligand of PD-1, and this interaction leads to immune evasion of tumors. In contrast, Foxp3, another TIL marker, was negatively correlated with PD-L1 in this study. Although high Foxp3 is theoretically associated with poor prognosis based on the fact that Foxp3+ TILs work to prevent the immune system from attacking tumor cells, some studies have actually reported high Foxp3 as a better prognostic factor, which suggests that the correlations of Foxp3 expression on TILs with PD-L1 expression on tumor cells are still controversial in terms of prognosis^14,30^.

Patil *et al*. recently reported a correlation between the expression of PD-L1, TILs, and mismatch repair (MMR) proteins and clinicopathologic findings in gastric adenocarcinomas in which PD-L1 was expressed in a large proportion (70%) of patients. They found that PD-L1 expression and MMR deficiency were associated with increases in TIL numbers and larger tumor size^31^. Although their study included more advanced cases than our study, Patil *et al*. found higher PD-L1 positivity (70%) compared with our study (15.5%). This could have been caused by differences in evaluation criteria, including the cutoff value of >1% in their study versus >5% in our study, and the type of PD-L1 antibody used. Patil *et al*. reported no significant difference between PD-L1 positivity and survival, which could have been related to the large population of PD-L1-positive tumors, whereas we found no significant difference when the PD-L1 cutoff value was set at 1%.

While this study provides some important information with potential impact in clinical practice, it has several limitations. First, it was a retrospective, single-center study. Second, many patients with early stage gastric cancer, who are unlikely to die of gastric cancer, were included in the OS analysis. When patients with only advanced (Stage II or higher) gastric cancer were analyzed, the influence of PD-L1 expression on prognosis was actually estimated smaller. Third, four mononucleotide repeat microsatellite targets of BAT26, NR27, NR21, and CAT25 were used for MSI analysis in this study, according to previously described protocols^32,33^. However, MLH1, MSH2, MSH6, and PMS2 are the most commonly used target markers in MSI analyses.

In conclusion, we demonstrated that PD-L1 overexpression on tumor cells was an independent prognostic factor in gastric cancer, and moreover, combination of PD-L1 with MSI or CD8 was a stronger prognostic marker than a single marker of PD-L1. Evaluation of MSI status and CD8+ TILs in addition to PD-L1 expression on tumor cells is considered important from the perspective of assessing the condition of the tumor microenvironment based on the immunity cycle, which is expected to lead to the development of more appropriate treatment strategies for gastric cancer.

## Materials and Methods

All experiments were conducted in accordance with the relevant guidelines and regulations of Okayama University.

### Patients

The medical records of 283 patients with gastric cancer who underwent gastrectomy at Okayama University Hospital (Okayama Prefecture, Japan) between 2002 and 2009 were reviewed retrospectively. Patient characteristics included age and sex. Histological type was classified into intestinal type and diffuse type, and status of lymphatic invasion (ly), venous invasion (v), tumor location, depth of tumor invasion (T), lymph node metastasis (N), and stage were described according to the 3rd English edition of the Japanese classification of gastric carcinoma^34^. This study was reviewed and approved by the institutional review board of Okayama University (No. 1505–023). Informed consent was obtained by the opt-out method.

### Immunohistochemistry

Formalin-fixed, paraffin-embedded (FFPE) tissue samples cut at 4 μm were deparaffinized in xylene and rehydrated in a graded ethanol series. After blocking endogenous peroxidases by incubation with 3% H_2_O_2_ for 10 min, the samples were boiled in citrate buffer (pH 6.0) for 14 min in a microwave oven for antigen retrieval. The samples were incubated with primary antibodies overnight at 4 °C and then with peroxidase-linked secondary antibody for 30 min at room temperature. After 3, 3-diaminobenzidine (DAB) staining for signal generation and counterstaining with Mayer’s hematoxylin, the samples were dehydrated and mounted onto coverslips. Antibodies to human PD-L1 (E1L3N; Cell Signaling Technology, Danvers, MA, USA), CD8, CD4, Foxp3, and PD-1 were used.

### Classification of PD-L1

**Figure 4:**
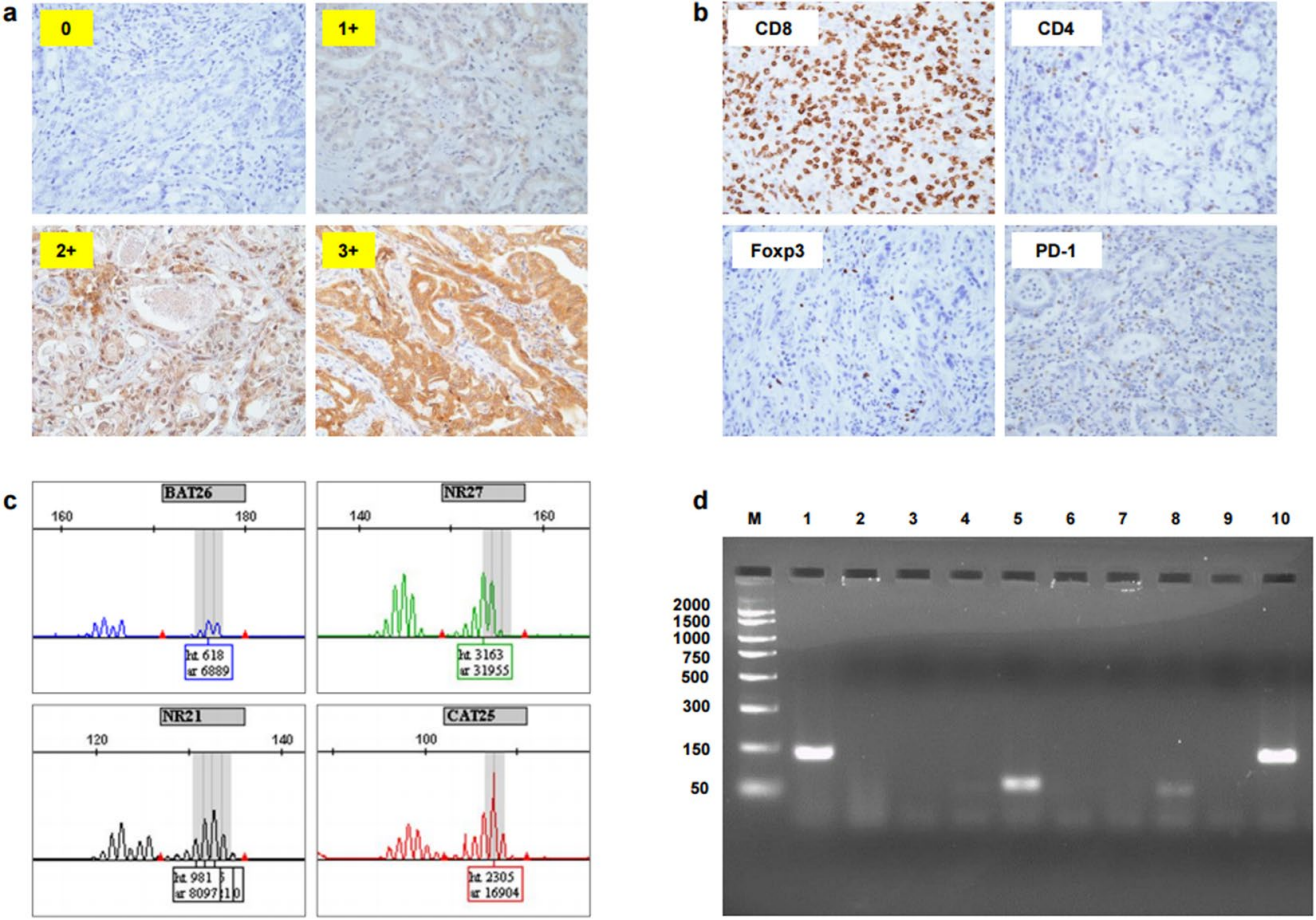
Representative pictures and data of each PD-L1 expression level (0, 1+, 2+ and 3+) (**a**), immunohistochemical staining for CD8, CD4, Foxp3, and PD-1 (**b**), four mononucleotide repeat microsatellite targets (BAT26, NR27, NR21 and CAT25) (**c**) and electrophoresis for EBV (**d**). The original gel was presented in Supplementary Fig. S3.

PD-L1 expression was classified into four groups according to PD-L1-positive rate on tumor cells, as determined by immunohistochemical staining (0: <1%, 1+: 1–5%, 2+: 5–10%, 3+: ≥10%) (Fig. 4a), and 2+ and 3+ were defined as PD-L1-positive. The evaluation was blindly performed by a pathologist.

### Evaluation of TILs

The average number of TILs with expression of CD8, CD4, Foxp3, or PD-1 (Fig. 4b) was calculated from three different randomly selected fields and classified into two groups (positive and negative) based on a cutoff value of 20 for CD8, 1 for CD4, 1 for Foxp3, and 1 for PD-1.

### Analysis of MSI

Genomic DNA was extracted from FFPE gastric cancer tissues using TaKaRa DEXPAT (Takara Bio Inc., Shiga, Japan) following separation of tumor and normal tissue by manual microdissection. MSI was evaluated with polymerase chain reaction (PCR) using primers for four mononucleotide repeat microsatellite targets (BAT26, NR27, NR21, and CAT25) according to a previously described protocol (Fig. 4c)^32,33^. Tumors showing allelic shifts in ≥two of four markers were defined as MSI-high (hereafter referred to as MSI) and the rest were defined as microsatellite stable (MSS, hereafter referred to as non-MSI).

### Detection of EBV

Genomic DNA collected from FFPE tissues was subjected to PCR using EBV primers^35^. The reaction mixture was fractionated electrophoretically on a 3% agarose gel and the DNA bands were visualized under UV light (Fig. 4d).

### Statistical analysis

Statistical analysis was conducted using JMP software (SAS Institute, Cary, NC, USA) by a different researcher from the ones who performed the staining and evaluation. Student’s *t*-test was used to assess the continuous variable of age, and the Wilcoxon signed-rank test was used for continuous variables of the number of lymphocytes expressing CD8, CD4, Foxp3, and PD-1. Pearson’s chi-square test was used for categorical variables of sex, histological type, ly, v, tumor location, T status, N status, stage, MSI, and EBV. The log-rank test was used for Kaplan–Meier survival analysis. Univariate and multivariate Cox proportional hazards regression analyses were performed to assess the effects of the prognostic factors.

## Supplementary information

Supplementary Information

Supplementary Table S1
