# Supplementary material for: PD-L1 expression combined with microsatellite instability/CD8+ tumor infiltrating lymphocytes as a useful prognostic biomarker in gastric cancer
Source: Sci Rep. 2019 Mar 15;9:4633. doi: 10.1038/s41598-019-41177-2 (PMC6420501; doi:10.1038/s41598-019-41177-2)
Supplement: Supplementary file 1 — Supplementary Information [file 41598_2019_41177_MOESM1_ESM.pdf]

Supplementary Information

PD-L1 expression combined with microsatellite instability/CD8+ tumor infiltrating lymphocytes as a useful prognostic biomarker in gastric cancer

Toshiaki Morihiro, Shinji Kuroda, Nobuhiko Kanaya, Yoshihiko Kakiuchi,
Tetsushi Kubota, Katsuyuki Aoyama, Takehiro Tanaka, Satoru Kikuchi,
Takeshi Nagasaka, Masahiko Nishizaki, Shunsuke Kagawa, Hiroshi Tazawa,
Toshiyoshi Fujiwara

Supplementary Table S1. PD-L1 expression on primary tumor and metastatic lymph nodes.

Supplementary Figure S1. Kaplan–Meier survival curve of gastric cancer patients based on PD-L1 expression on primary tumors and metastatic lymph nodes.

Supplementary Figure S2. Kaplan–Meier survival curve of gastric cancer patients based on the combined marker of PD-L1 expression on primary tumors and metastatic lymph nodes.

Supplementary Figure S3. Original gel used in the main figure (Fig. 4d).

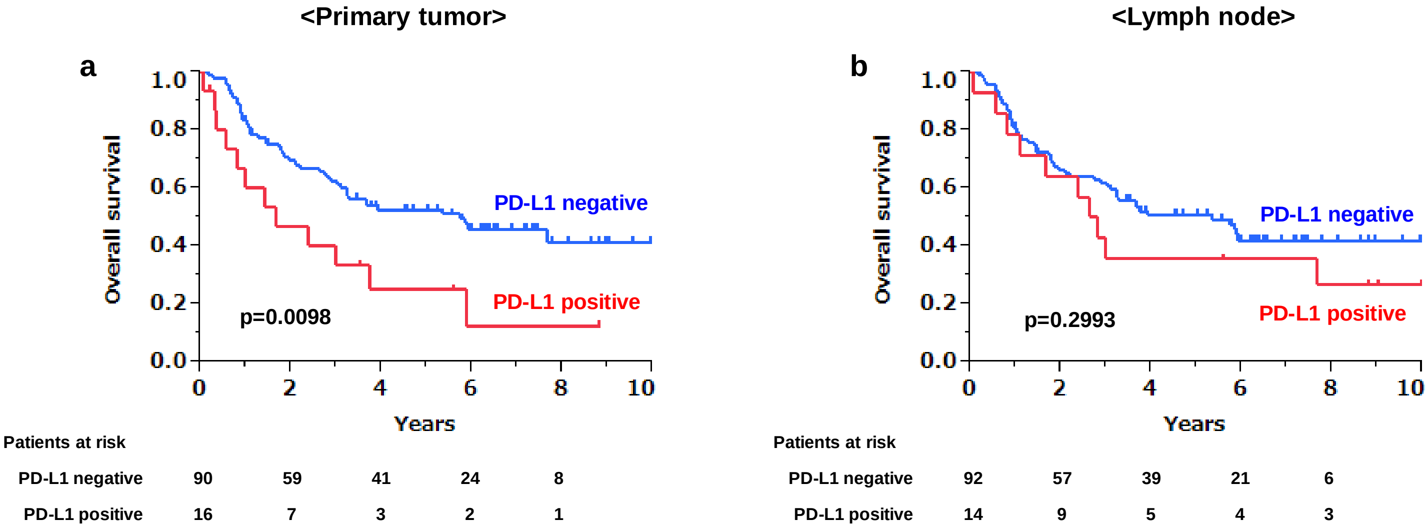

Supplementary Figure S1. Kaplan–Meier survival curve of gastric cancer patients based on PD-L1 expression on primary tumors (a) and metastatic lymph nodes (b).

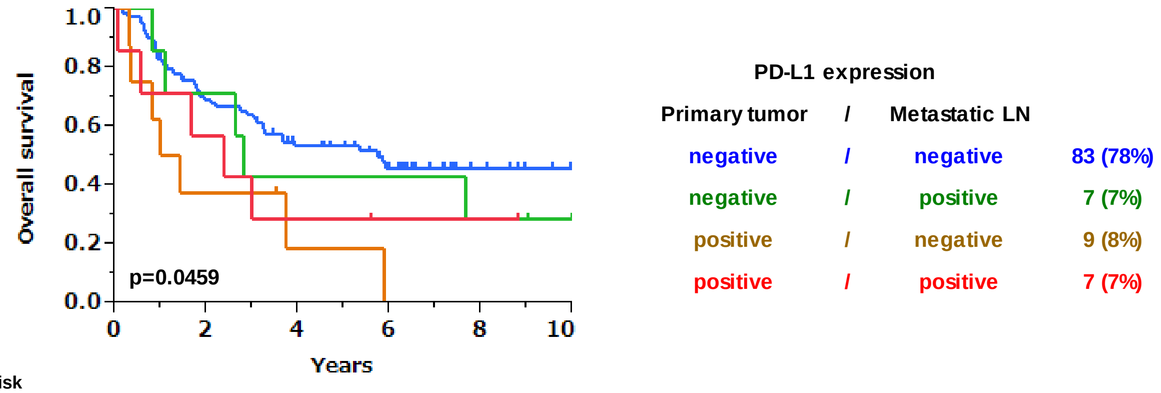

Patients at risk

negative / negative	83	54	38	21	6
negative / positive	7	5	3	3	2
positive / negative	9	3	1	-	-
positive / positive	7	4	2	1	1

Supplementary Figure S2. Kaplan–Meier survival curve of gastric cancer patients based on the combined marker of PD-L1 expression on primary tumors and metastatic lymph nodes.

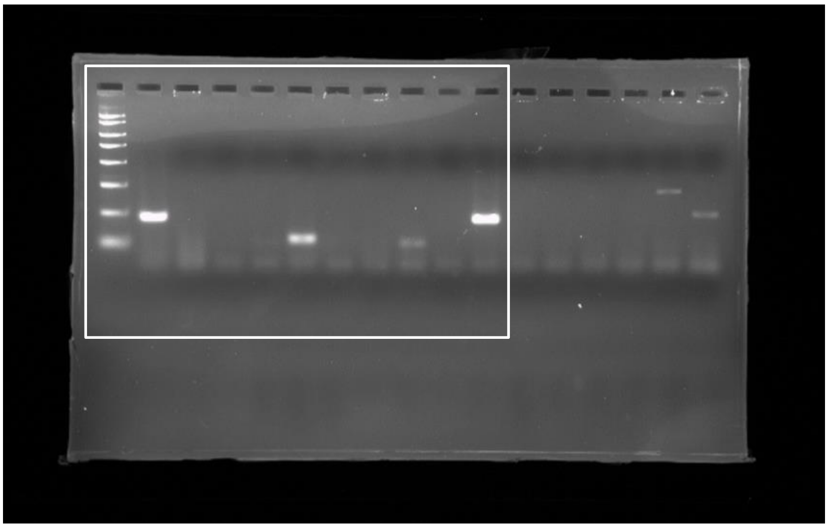

Supplementary Figure S3. Original gel.

Area surrounded by white line was used in the main figure (Fig. 4d).
